# The Relationship Between Refeeding Syndrome and Preterm Morbidities in Preterm Infants

**DOI:** 10.3390/children12101370

**Published:** 2025-10-10

**Authors:** Aybuke Yazici, Ipek Guney Varal, Gaffari Tunc, Onur Bagci, Ayse Oren

**Affiliations:** Division of Neonatology, Department of Pediatrics, Bursa Yüksek İhtisas Training and Research Hospital, University of Health Sciences, 16310 Bursa, Turkey; ipek.guneyvaral@sbu.edu.tr (I.G.V.); gaffari.tunc@saglik.gov.tr (G.T.); onur.bagci1@saglik.gov.tr (O.B.); ayse.oren@sbu.edu.tr (A.O.)

**Keywords:** preterm, refeeding syndrome, respiratory distress syndrome, morbidity

## Abstract

**Highlights:**

**What are the main findings?**

- The findings suggest that RFS in preterm infants is associated with gestational immaturity, low birth weight, perinatal compromise, and SGA status, and correlates with increased respiratory distres sydrome and prolonged hospitalization.
- With more frequent laboratory assessment of infants with RDS, the necessary electrolyte support can be provided early, thereby reducing respiratory distress in these infants.

**What is the implication of the main finding?**

- These results highlight the importance of vigilant metabolic monitoring and preventive nutritional strategies for vulnerable preterm populations.
- The early identification of infants at risk and the careful titration of parenteral and enteral nutrition are crucial for minimising complications and improving outcomes.

**Abstract:**

**Objective**: This study was conducted to determine the risk factors for refeeding syndrome (RFS) in preterm infants and evaluate its relationship with preterm morbidities. **Methods**: Preterm infants born before 30 weeks of gestation were retrospectively evaluated. RFS was diagnosed as phosphorus <4 mg/dL and/or calcium >11 mg/dL on postnatal day 7. Demographic and clinical findings were compared between preterm infants with and without RFS. **Results**: A total of 174 infants who met the inclusion criteria were analyzed. RFS was diagnosed with 60 infants (34.5%). The mean gestational age (GA) was 27 (range, 25–28) weeks in the RFS group and 28 (range, 26–30) weeks in the non-RFS group (*p* = 0.038). Mean birth weight (BW) in each group was 790 (range, 630–1000) grams and 1033 (range, 800–1310) grams, respectively (*p* < 0.001). The RFS group had a lower rate of antenatal steroid (ANS) administration, lower APGAR, length of hospital stay, and higher rates of small for gestational age status, respiratory distress syndrome (RDS), bronchopulmonary dysplasia, and patent ductus arteriosus (*p* < 0.05). On postnatal day 7, the RFS group had lower phosphorus and higher calcium levels (*p* < 0.001). After adjustment for GA, BW, Apgar score, and ANS, the frequency of RDS was higher among infants with RFS (*p* < 0.05). **Conclusions**: Preterm infants with RDS were more likely to develop RFS. Our results suggest that these infants require more frequent laboratory testing and closer follow-up to monitor for RFS and ensure timely electrolyte support.

## 1. Introduction

Preterm infants are at high risk of malnutrition after birth. Not having completed the third trimester of gestation, they are born with insufficient electrolyte, fat, and glycogen stores. As a result, these infants require immediate enteral and parenteral nutrition [1]. A fetus needs 3–4 g/kg/day of protein for growth and brain development. However, preterm infants are exposed to a different environment from intrauterine life upon birth and require more energy to maintain thermal and metabolic homeostasis [2]. The upper limit of initial energy and protein required to support postnatal growth in extremely low birth weight (ELBW) infants is not clear. The enteral nutrition guideline published by the European Society of Pediatric Gastroenterology, Hepatology and Nutrition (ESPGHAN) in 2022 states that to mimic intrauterine life, protein intake of 4 g/kg/day and energy intake of at least 115–140 kcal/kg/day can support growth in preterm infants [3].

Although there is no clear definition of refeeding syndrome (RFS), it has been described as a condition seen in malnourished patients who administration aggressive nutrition and can result in hypophosphatemia, hypercalcemia, hypokalemia, hypernatremia, and hypomagnesemia [4,5,6,7]. The frequency of RFS and risk factors for RFS in ELBW infants remain uncertain, but it has been reported that high amino acid and energy intake without adequate electrolyte intake may lead to RFS [8]. In infants with RFS, the rise in insulin resulting from increased nutrition causes depletion of both intracellular and extracellular phosphorus, as well as intracellular influx of potassium and magnesium ions. Phosphorus plays critical roles in the formation of adenosine triphosphate, oxygen delivery to tissues, acid–base buffering, and the activation of many enzymes. Therefore, phosphorus depletion leads to cellular dysfunction [9]. Hypophosphatemia is included in the definition of RFS and can lead to life-threatening conditions if not treated [10]. Different phosphorus levels have been reported in the literature as cutoffs for hypophosphatemia [8].

Preterm infants are usually born after pregnancy with complications such as placental insufficiency [11]. Although phosphate is normally actively transferred across the placenta, the supply of phosphate to the fetus decreases with the development of placental insufficiency [12]. With the initiation of parenteral feeding after birth, the infant’s intracellular energy demand increases, resulting in an abrupt increase in phosphorus usage. Depletion of extracellular phosphorus triggers the resorption of calcium and phosphorus from the bone to ensure hemostasis, resulting in elevated calcium levels. Therefore, hypophosphatemia and hypercalcemia are included in the definition of RFS [7].

Preterm infant nutrition remains a challenge in clinical practice. Malnutrition in the first days of life can cause increased susceptibility to sepsis, severe respiratory distress, and various organ dysfunctions. Therefore, the biochemical abnormalities associated with RFS should be mitigated through careful monitoring and patient acclimation in the clinical management of parenteral and enteral nutrition. Our aim in this study was to determine the risk factors for RFS in preterm infants and evaluate RFS’ relationship with preterm morbidities to help ensure that clinicians take the necessary precautions in infants at risk of RFS.

## 2. Materials and Methods

Preterm infants that were born before 30 weeks of gestation and admitted to the level 4 neonatal intensive care unit of our hospital between January 2023 and May 2025 were evaluated retrospectively. Infants with congenital anomaly or metabolic disease and those who underwent surgery were excluded. Biochemical analyses were performed at admission (day 1) and on day 7. RFS was diagnosed as serum concentrations of phosphorus <4 mg/dL and/or calcium >11 mg/dL in laboratory values taken on day 7 [13]. Demographic and clinical data were compared between preterm infants with and without RFS.

Enteral and parenteral nutrition were administered in accordance with the recommendations of the Turkish Neonatal Society [14,15]. For enteral nutrition of preterm infants, early provision of breast milk was preferred. If breast milk was not available, preterm formula was used. Starting in the first hours, total parenteral nutrition (TPN) was initiated together with oral care with colostrum and minimal enteral nutrition. Expressed breast milk was given via catheter and increased while closely monitoring feeding tolerance. The amount of food was increased at a rate appropriate to the infant’s birth weight (BW)/gestational age (GA) and risk status, aiming for full enteral nutrition in the first week in infants over 1000 g and in the second week for infants below 1000 g. When enteral feeding volume reached 100 mL/kg, breast milk fortifier was added to the breast milk [14]. In TPN, amino acids were started at 3 g/kg/day on the first day and gradually increased over a few days to 4 g/kg/day for ELBW infants and 3.5 g/kg/day for very low birth weight (VLBW) infants. Sodium, potassium, and chlorine were added to TPN approximately 72 h after the patient diuresed. Calcium and phosphate (at a ratio of 1.7:1) were added to TPN starting on the first day [15]. Feeding intolerance was defined as a deterioration in clinical condition, abnormal abdominal examination findings, vomiting, >50% and/or bloody residual (if examined), and change in stool frequency [14].

Sepsis was defined according to the European Medicines Agency (EMA) sepsis scoring system [16]. Late-onset neonatal sepsis was defined as the onset of symptoms at least 72 h after birth [17]. The oxygen requirement of patients with bronchopulmonary dysplasia (BPD) was evaluated and classified at postnatal day 28, postmenstrual week 36, and discharged [18]. Respiratory support was determined according to the standard respiratory support protocol of our unit, and surfactant was administered to patients with respiratory distress syndrome (RDS) [19]. Cranial ultrasound was performed by a certified neonatologist on days 1, 3, and 7, and weekly thereafter depending on signs of intraventricular hemorrhage (IVH). IVH was graded according to the Volpe classification [20]. Patent ductus arteriosus (PDA) was diagnosed according to echocardiography performed by a pediatric cardiologist at postnatal 48 h [21]. Consent to conduct the study was obtained from the local ethics committee (ethics committee no: 2024-TBEK 2025/07-09).

### Statistical Analysis

Histograms, Q-Q plots, and Shapiro–Wilk test were used to assess the normality of data distributions. The Levene test was used to test the homogeneity of variances. To compare demographic, clinical, and laboratory parameters between patients with and without RFS, a two-sided independent samples *t* test or the Mann–Whitney U test was used for continuous variables, while Pearson chi-square analysis or the Fisher-Freeman-Halton test was used for categorical variables. Covariance analyses were conducted to make the same comparisons while correcting for the effect of GA, BW, and 5-min Apgar score. A two-sided paired *t* test or Wilcoxon *t* tests were used for within-group comparisons of laboratory values.

## 3. Results

**Figure 1:**
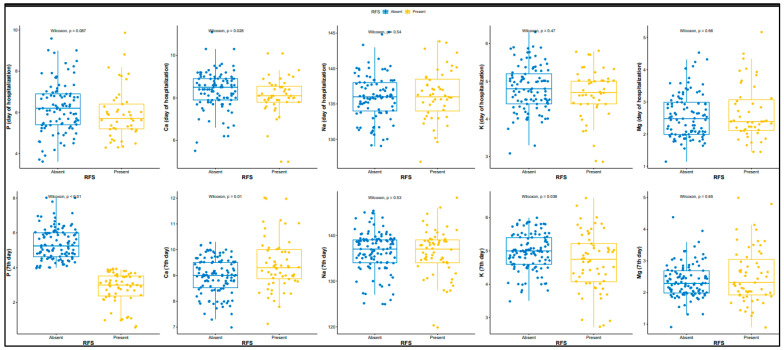
Changes in serum phosphate, calcium, sodium, potassium and magnesium from admission to day 7 in patients with versus without refeeding syndrome. (Blue color: RFS absent, yellow color: RFS present).

The records of 296 preterm infants hospitalized in our unit during the study period were screened. After applying the inclusion and exclusion criteria, 174 preterm infants were included in the analysis. RFS was diagnosed with 60 infants (34.5%). The mean GA was 27 (range, 25–28) weeks in the RFS group and 28 (range, 26–30) weeks in the non-RFS group (*p* = 0.038). Mean BW in each group was 790 (range, 630–1000) grams and 1033 (range, 800–1310) grams, respectively (*p* < 0.001). The RFS group had a lower rate of antenatal steroid use (*p* = 0.04), lower Apgar scores at 1 and 5 min (*p* = 0.01 and *p* = 0.02, respectively), and were more likely to be small for gestational age (SGA) (*p* = 0.015) (Table 1). The RFS group also had higher rates of RDS (*p* = 0.001), BPD (*p* = 0.04), and PDA (*p* = 0.01) and longer length of hospital stay (*p* < 0.01) (Table 2). Phosphorus and calcium levels at admission were similar between the two groups (*p* = 0.08, *p* = 0.06, respectively), whereas on day 7 the RFS group had a lower phosphorus level (*p* < 0.001) and higher calcium level (*p* < 0.001) (Table 3) (Figure 1). In adjusted analysis using GA, BW, Apgar score, and antenatal steroid use as correction factors, the frequency of RDS was higher among infants with RFS (*p* = 0.04) (Table 2).

## 4. Discussion

According to the results of our study, infants with RFS had lower GA and BW, with higher rates of SGA and RDS. After adjustment for GA, BW, Apgar score, and antenatal steroid use, the prevalence of RDS was found to be higher in infants with RFS. This finding suggests that in patients with RDS, the respiratory workload could be reduced by performing laboratory tests to monitor for RFS more frequently and providing the necessary electrolyte support.

The provision of adequate amounts of protein and energy to preterm infants is essential for their growth, as it promotes tissue anabolism. In the rapidly growing preterm infant, 0.3 mmol phosphate is required for each gram of protein used for tissue growth [22]. In one study, hypophosphatemia was detected in the first week of life in 94% of preterm infants weighing less than 1000 g and 61% of those weighing more than 1000 g. Infants with hypophosphatemia were found to have a lower GA and BW [23]. In another study in which 38% of infants born before 32 weeks’ gestation developed RFS, lower GA and BW were also noted in those who developed RFS [24]. Similarly to these two studies, GA and BW were lower in the infants with RFS in our study, and the prevalence

Factors such as preeclampsia, increased resistance in the umbilical artery, and intrauterine growth restriction have been associated with RFS [25,26]. Evaluation of electrolyte levels during the first 5 days of life in infants at 24–27 weeks’ gestation who had been administered with high lipids and amino acids from birth showed that hypokalemia and hypophosphatemia were more common in SGA infants [27]. Based on these findings, the researchers stated that the parenteral nutrition strategy currently recommended increases the risk of early hypokalemia and hypophosphatemia in SGA infants and emphasized that potassium and phosphorus levels in these infants should be adjusted to adequate levels from birth. Similarly, RFS was more frequent among SGA infants in the present study. In a study comparing ELBW infants who initially received two different initial amino acid protocols, low (1.5 g/kg/day) or high (3 g/kg/day), the prevalence of hypophosphatemia and severe hypophosphatemia increased from 51% and 8% with low amino acids to 59% and 20% with high amino acids, respectively. It was observed that serum phosphorus levels reached the lowest level on the postnatal day 6. The authors concluded that high initial amino acid intake significantly increased the risk of severe hypophosphatemia and RFS in ELBW infants and recommended a parenteral nutrition protocol with initial low energy intake gradually increasing over the first week for SGA ELBW infants [28]. Another study testing the impact of adding 1 g of amino acids per day for the first 5 days after birth in ELBW infants revealed no significant difference in the incidence of survival without neurodisability at 2 years, whereas the rate of RFS increased (24.4% vs. 15.6%). Based on these findings, high protein administration was not considered necessary to support growth [29]. Moltu et al. also administered higher protein and energy to ELBW infants in their intervention group to determine the effect on growth and cognitive development compared to the control group. Although postpartum growth was improved in the intervention group, hypophosphatemia, hypokalemia, and hypercalcemia were more frequent during the first week, leading to the conclusion that fortified nutrition causes electrolyte imbalance in VLBW infants [30].

In a study evaluating risk factors for the development of hypophosphatemia, Yakubovich et al. measured phosphate levels on postnatal days 3 and 7–10 and determined that delayed enteral nutrition (>3 days) was associated with lower serum phosphorus levels. Additionally, serum phosphorus levels were significantly higher in the formula-fed group (5.90 ± 1.48) than in the group without enteral nutrition (4.78 ± 1.6) (*p* < 0.001) and the breast milk-fed group (5.3 ± 1.15) (*p* = 0.027) [31]. In our study, no relationship was found between RFS and type of nutrition or feeding intolerance. Yakubovich et al. also reported that although serum phosphorus level on day 3 did not help predict morbidity or mortality, significant results were obtained for day 7 phosphorus. However, after adjusting for confounders such as GA, sex, and maternal hypertension, serum phosphorus level at either time point was unable to predict BPD, necrotizing enterocolitis, IVH, late-onset neonatal sepsis, or mortality [31]. In the present study, electrolyte values on postnatal day 7 were used to define RFS. When GA, BW, Apgar score, and antenatal steroid use were adjusted for, the incidence of RDS was found to be significantly higher among infants with RFS.

Red blood cells contain 2,3-diphosphoglycerate (2,3-DPG), an important component that regulates oxygen release from hemoglobin. In hypophosphatemia, the decrease in inorganic phosphate concentration in the red blood cells results in reduced 2,3-DPG synthesis [32]. In a study similar to ours, hypophosphatemia developing in the first week of life was found to be associated with RDS, and phosphorus deficiency was proposed as a marker of disease severity in infants with RDS [23]. The greater energy demands of infants with RDS are believed to increase phosphorus use and lead to hypophosphatemia. Infants weighing <1250 g treated with high calories and protein were reported to have a higher risk of hypophosphatemia (serum phosphorus <4 mg/dL) and longer duration of mechanical ventilation [33]. In that study, infants who developed hypophosphatemia had lower GA and BW than those who did not. Similarly, despite correcting for GA and BW in our study, the rate of RDS was higher among hypophosphatemic infants. As serum phosphate is not routinely checked at birth in VLBW infants, the frequency of hypophosphatemia and RFS in these infants is not clearly known [34]. These results suggests that serum phosphorus levels should be screened early in infants being treated for RDS, which would allow for timely phosphorus support and possibly contribute to preventing the worsening of RDS symptoms due to hypophosphatemia.

In a study conducted by Ross et al., BPD was observed more frequently in infants who developed RFS. The authors stated that the higher incidence of BPD in VLBW infants with hypophosphatemia may be attributable to the increased volutrauma and barotrauma occurring with prolonged mechanical ventilation [25]. In our study, although BPD was more common among infants with RFS, this significance was lost after adjusting for GA and BW. However, we may have observed a decrease in BPD as a result of our early identification of electrolyte deficiencies and provision of electrolyte support.

Limitations of this study are that it was a single-center, retrospective study without daily information about the infants’ enteral and parenteral nutrition. Furthermore, neurological outcomes and auxological parameters were not assessed during discharge, which limited the evaluation of long-term growth and developmental outcomes. Another limitation is that we did not perform more frequent sampling, only analyzing laboratory values on days 1 and 7. However, the inclusion of infants treated by the same clinicians with the same protocol over the last 2.5 years is a strength of our study.

## 5. Conclusions

Nutrition for preterm infants in Türkiye is provided in accordance with national guidelines. In TPN, amino acids are initiated at 2–3 g/kg/day on the first day and gradually increased over several days to 3.5–4 g/kg/day in ELBW infants and 3–3.5 g/kg/day in VLBW infants. Considering the evidence that RFS occurs with high protein administration in ELBW infants, prospective studies comparing low and high protein protocols are required to prove the accuracy of this hypothesis. A reduced number of tests are performed in preterm infants in order to avoid complications such as iatrogenic anemia and sepsis associated with blood sampling. According to the results of our study, RFS was more common in infants who developed RDS. As RFS may occur more frequently in infants with RDS and SGA, it may be clinically beneficial to assess these patients’ biochemical values in the first week.

## Figures and Tables

**Table 1:** Comparison of demographic variables in preterm infants with and without refeeding syndrome.

**Variable**	**Refeeding Syndrome**		**Total** **(*n* = 174)**	***p*-Value**
	**Absent** **(*n* = 114)**	**Present** **(*n* = 60)**		
Gestational age (weeks)	**28 (26–30)**	27 (25–28)	27 (25–29)	**0.03**
Birth weight (g)	1033 (800–1310)	790 (630–1000)	930 (705–1190)	**<0.001**
Maternal age (years)	27(22–32)	30 (25–35)	28 (23–32)	**0.04**
Cesarean birth	105 (92.9)	60 (100.0)	165 (95.4)	0.05
Need for resuscitation	40 (35.4)	26 (43.3)	66 (38.2)	0.3
Multiple pregnancy	24 (21.2)	12 (20.0)	36 (20.8)	0.84
Preeclampsia	12 (10.6)	11 (18.3)	23 (13.3)	0.15
Clinical chorioamnionitis	9 (8.0)	3 (5.0)	12 (6.9)	0.54
SGA	27 (23.9)	25 (41.7)	52 (30.1)	**0.015**
Maternal smoking	2 (1.8)	2 (3.3)	4 (2.3)	0.61
PDA	2 (1.8)	0 (0.0)	2 (1.2)	0.54
ANS	69 (60.5)	27 (45.0)	96 (55.2)	**0.04**
1-min Apgar score	6 (5–7)	5 (5–6)	6 (5–7)	**0.01**
5-min Apgar score	8 (7–9)	7 (6–8)	8 (7–8)	**0.02**

SGA: Small for gestational age, PDA: Patent ductus arteriosus, ANS: Antenatal steroid. Significant *p*-values are shown in bold.

**Table 2:** Comparison of clinical variables in preterm infants with and without refeeding syndrome.

**Variable**	**Refeeding Syndrome**		**Total** **(*n* = 174)**	***p*-Value**	**adj. *p* Value**
	**Absent** **(*n* = 114)**	**Present** **(*n* = 60)**			
RDS	**72 (63.2)**	**52 (86.7)**	124 (71.3)	**0.001**	**0.04**
BPD	58 (50.9)	40 (66.7)	98 (56.3)	**0.04**	0.45
PDA	52 (45.6)	39 (66.1)	91 (52.6)	**0.01**	0.18
Enteral nutrition	3 (2–4)	3 (2–4)	3 (2–4)	0.56	0.05
Type of nutrition					
Breast milk (BM)	90 (81.8)	37 (69.8)	127 (77.9)	0.22	0.95
Preterm formula (PF)	15 (13.6)	12 (22.6)	27 (16.6)		
Mixed (BM + PF)	5 (4.5)	4 (7.5)	9 (5.5)		
TPN (days)	18 (10–31)	22 (15–37)	17 (10–30)	0.07	0.38
Day of full enteral nutrition (120 kcal/kg/day)	21 (11–45)	30 (16–49)	22 (10–43)	0.05	0.89
Feeding intolerance	70 (61.4)	43 (71.7)	114 (64.9)	0.17	0.68
Feeding intolerance in first 72 h	62 (54.4)	40 (66.7)	102 (58.6)	0.11	0.82
LOS	73 (64)	42 (70)	115 (66)	0.29	0.59
IVH (grade 2–3)	12 (10.5)	11 (18.3)	23 (13.2)	0.14	0.41
Length of hospital stay (days)	58 (41–83)	91 (58–113)	68 (48–94)	**<0.001**	**0.04**

adj. *p*-value: Adjusted for gestational age, birth weight, and 5-min Apgar score. RDS: Respiratory distress syndrome, BPD: Bronchopulmonary dysplasia, PDA: Patent ductus arteriosus, TPN: Total parenteral nutrition, LOS: Late-onset sepsis, IVH: Intraventricular hemorrhage. Significant *p*-values are shown in bold.

**Table 3:** Comparison of laboratory variables in preterm infants with and without refeeding syndrome at admission (day 1) and on day 7.

**Variable**	**Refeeding Syndrome**		**Total** **(*n* = 174)**	***p*-Value ^†^**	**adj. *p*-Value ^†^**
	**Absent** **(*n* = 114)**	**Present** **(*n* = 60)**			
Phosphorus (mg/dL)					
Day 1	6.20 (5.40–6.90)	5.70 (5.20–6.40)	6.00 (5.20–6.80)	0.08	0.44
Day 7	5.25 (4.60–6.00)	3.00 (2.35–3.55)	4.60 (3.50–5.70)	**<0.001**	**<0.001**
*p*-value ^‡^	**<0.001**	**<0.001**	**<0.001**		
Calcium (mg/dL)					
Day 1	8.37 ± 0.90	8.07 ± 0.96	8.28 ± 0.89	0.06	0.06
Day 7	8.98 ± 0.71	9.44 ± 1.02	9.14 ± 0.85	**0.002**	**0.001**
*p*-value ^‡^	**<0.001**	**<0.001**	**<0.001**		
Magnesium (mg/dL)					
Day 1	2.48 (1.99–2.99)	2.39 (2.10–3.11)	2.44 (2.02–3.05)	0.65	0.39
Day 7	2.29 (1.99–2.69)	2.32 (1.91–3.12)	2.30 (1.98–2.75)	0.65	0.25
*p*-value ^‡^	**0.023**	0.552	**0.029**		
Sodium (mmol/L)					
Day 1	135.94 ± 3.13	136.30 ± 3.64	136.14 ± 3.37	0.54	0.78
Day 7	136.61 ± 4.40	136.10 ± 5.29	136.44 ± 4.71	0.49	0.99
*p*-value ^‡^	0.339	0.654	0.296		
Potassium (mmol/L)					
Day 1	4.81 ± 0.61	4.67 ± 0.68	4.76 ± 0.64	0.24	0.99
Day 7	4.96 ± 0.55	4.69 ± 0.88	4.87 ± 0.70	**0.03**	**0.03**
*p*-value ^‡^	**0.036**	0.184	**0.015**		

*p*-value ^†^: Significance value for between-group comparisons. *p*-value ^‡^: Significance value for within-group comparisons. adj. *p*-value: Adjusted for gestational age, birth weight, and 5-min Apgar score. Significant *p*-values are shown in bold.

## Data Availability

The original contributions presented in this study are included in the article. Further inquiries can be directed to the corresponding author.

## Abbreviations

RFS	refeeding syndrome
GA	gestational age
BW	birth weight
ANS	antenatal steroid
RDS	respiratory distress syndrome
ELBW	extremely low birth weight
ESPGHAN	European Society of Pediatric Gastroenterology, Hepatology and Nutrition
TPN	total parenteral nutrition
VLBW	very low birth weight
BPD	bronchopulmonary dysplasia
IVH	intraventricular hemorrhage
PDA	patent ductus arteriosus
SGA	small for gestational age
